# Unique Case of Parathyroid Adenoma With Arteriovenous Malformation

**DOI:** 10.7759/cureus.41206

**Published:** 2023-06-30

**Authors:** Abdalla Saad Abdalla Al-Zawi, Ahmed Shah, Amira Asaad, Saman Jalilzadeh Afshari, Salem Alowami

**Affiliations:** 1 General & Breast Surgery, Mid and South Essex University Hospital Group, Basildon, GBR; 2 General & Breast Surgery, Basildon and Thurrock University Hospital, Basildon, GBR; 3 General & Breast Surgery, Anglia Ruskin University, Chelmsford, GBR; 4 Pathology and Molecular Medicine, McMaster University, Hamilton, CAN; 5 Surgery, University College Hospital, London, GBR; 6 Surgery, Oxford University Hospitals NHS Foundation Trust, Oxford, GBR; 7 Pathology, McMaster University, Hamilton, CAN

**Keywords:** osler-weber-rendu disease, wyburn-mason syndrome, arteriovenous malformation, hyperparathyroidism, parathyroid adenoma

## Abstract

Direct communication between dysmorphic arteries and veins without an interceding capillary segment is known as arteriovenous malformation (AVM). Its etiology is still unknown; however, it is commonly acknowledged that it could be related to trauma or is congenital in origin. Often, AVMs are found in the central nervous system or other sites such as under the skin or in the deep solid organs. They can be encountered as a solitary abnormality or associated with another pathology. If they are large enough, they can deprive the neighboring tissue of oxygen, eventually leading to tissue damage and compressing the surrounding organs, causing potentially more serious consequences. AVM in parathyroid adenoma is an unusual entity in the medical reports and known clinical practice. We herein report a unique case of a 49-year-old female patient who presented with a neck mass and associated symptoms of hyperparathyroidism (HPT) with no history of previous trauma or surgery. The imaging and laboratory tests were consistent with parathyroid neoplasm. Parathyroidectomy was performed and revealed parathyroid adenoma with AVM.

## Introduction

Parathyroid adenomas are usually functional and manifest clinically with hypercalcemia; however, they may be detected as a non-functional lesion [1]. They are well-known to be associated with generalized skeletal demineralization, widespread cystic bone disease, and recurrent renal calculi [1]. They may be associated with certain abnormalities, such as ectopic parathyroids, in the mediastinum [2]. Arteriovenous malformation (AVM) is a condition in which there is a direct link between arteries and veins, bypassing the normal intervening capillary bed. Their pathogenesis could be related to inherited factors or trauma such as needle biopsy. To the best of our knowledge, this paper describes the first case in the medical literature of a patient with a functional parathyroid adenoma associated with arteriovenous malformation.

## Case presentation

**Figure 1:**
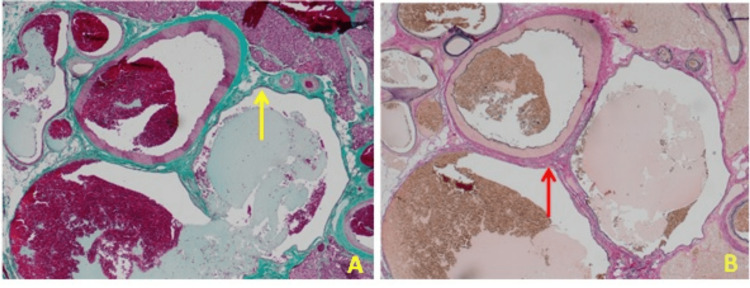
(A) Trichrome stain showing the vessels' wall (yellow arrow), x40; (B) Miller's stain showing elastic lamina in vessels wall (red arrow), x40

We report a case of a 49-year-old woman who presented with symptoms of hyperparathyroidism (HPT) and a neck mass. The patient’s technetium (99mTc) sestamibi report showed uptake of the right inferior parathyroid. Her blood work revealed elevated parathyroid hormone (PTH) of 36.7 pmol/L, high total calcium of 2.55 mmol/L, and ionized calcium of 1.49 mmol/L. The patient had a normal thyroid-stimulating hormone (TSH) of 2.15 mIU/L and a low vitamin D level of 36.7 nmol/L. The CT scan of the neck showed an ovoid right inferior parathyroid mass measuring 2.2 x 1.0 x 0.8 cm just inferior to the right thyroid lobe at the neck base, extending caudally to the level of the jugulum. The mass demonstrated central cystic changes. The CT scan did not reveal cervical lymphadenopathy or lytic or sclerotic bony lesions. Overall, this was compatible with a right inferior parathyroid adenoma. Subsequently, the patient underwent a parathyroidectomy of her enlarged left inferior parathyroid adenoma. Postoperatively, the patient’s calcium was normal, and her PTH lowered to 6.2 pmol/L; therefore, she was discharged without complications. On gross pathology, the specimen was a single brown, firm nodule measuring 2.8 x 1.5 x 1.3 cm and weighing 2.0 g. Histologically, the lesion was diagnosed as a hypercellular parathyroid gland in keeping with parathyroid adenoma, with numerous prominent vessels showing different wall thicknesses suggestive of arteriovenous malformation (Figure 1).

Further staining delineated collagen fibers in the vessel walls by using trichrome, which is an acidic dye that stains collagen blue (Figure 1A). The vascular elastic lamina was also clearly demonstrated using Miller's stain, which stains elastic fibers in black and collagen in red (Figure 1B).

## Discussion

Parathyroid adenoma is regarded as the most frequent parathyroid gland pathology causing symptomatic disease, accounting for 80-85% of cases [2]. Fuller Albright and Edward Reifenstein first reported on HPT in the 1930s [3]. Primary hyperparathyroidism (PHPT) is the third most common endocrine disorder after diabetes mellitus and thyroid diseases, and its incidence is highest between the fifth and sixth decades of life. It is observed to be two to three times more common in females than males [4]. About 90% of PHPT cases are sporadic, and 10% are familial. The known familial conditions include MEN1 (multiple endocrine neoplasia type 1), MEN2, MEN4, and HPT-JT (hyperparathyroidism-jaw tumor syndrome) [5]. It presents as a cervical lump with symptoms of HPT such as recurrent renal calculi and gastrointestinal, psychological, and skeletal symptoms (Table 1).

**Table 1:** Manifestations of hyperparathyroidism Sources: [1-6]

System	Manifestations
Skeletal	Bone pain, fracture
Renal	Nephrolithiasis, hypercalciuria
Gastrointestinal	Anorexia, nausea, vomiting, constipation, peptic ulcer, acute pancreatitis
Neuromuscular	Weakness, fatigue, intellectual disturbances, mental confusion, coma
Cardiovascular	Hypertension, shortened QT interval

**Figure 2:**
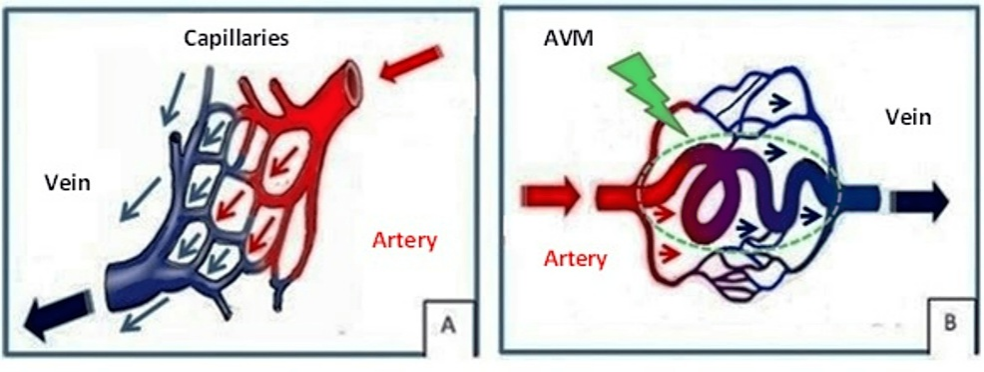
(A) Normal vasculature pattern. (B) Arteriovenous malformation AVM: arteriovenous malformation Image created by author Abdalla Saad Abdalla Al-Zawi.

Parathyroid adenoma is known as an isolated gland disease with a solitary adenoma nodule. Most of the lesions are solid in nature. Morphologically, they are seen as a well-circumscribed lesion, often surrounded by a fibrous capsule. The adenoma is composed of a single cell type - chief cells with round nuclei and scanty granular cytoplasm. There is little, if any, extracellular or intracellular fat component. Compressed normal parathyroid tissue may be seen at the periphery. Some adenomas occasionally may show follicular, acinar, pseudo-glandular, fibrous, or partially cystic patterns [5]. AVMs are high-flow vascular lesions characterized by direct communication between arteries and veins in the absence of a normal capillary bed in between (Figure 2) [6].

**Figure 3:**
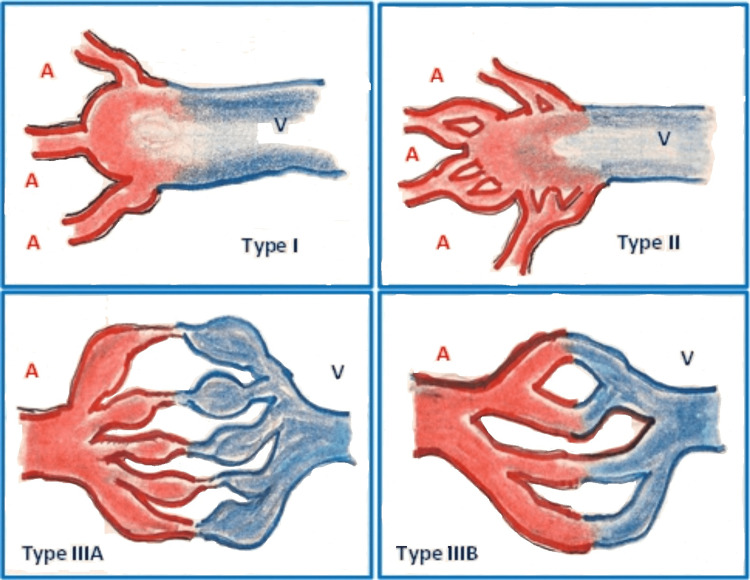
Cho classification for peripheral arteriovenous malformation A: artery; V: vein The illustration has been adapted from Neto et al. (2019) [18]; image redrawn by author Abdalla Saad Abdalla Al-Zawi.

The pathogenesis mechanisms of AVM are unknown; however, it has been reported that hereditary factors, trauma, ischemia related to thrombosis, and hormonal abnormalities can trigger AVM formation [6,7]. In this paper, in spite of the uniqueness of this case, as there are no records of AVM encountered in parathyroid adenoma, we can say that there are a few discussed aspects that are in line with the current known evidence. The current literature shows that AVMs and tumors can occur anywhere in the body. They may replace the host organ and alter its physiological function or behave as a space-occupying lesion and, if they are intraosseous, may cause bone weakness [7,8]. Worldwide, breast cancer is the most frequent type of cancer in females, and hormonal manipulation therapy is used widely as part of the management pathway. Some reports do show an effect of endocrine manipulation therapy with estrogen receptor antagonists or aromatase inhibitors on arteriovenous malformations [9,10]. AVM has been detected in the eyes, uterus, brain, small bowel, thyroid gland, limbs, liver, spleen, spine, lungs, and colon [11-13]. There are some conditions associated with AVM, such as Wyburn-Mason syndrome, which is a non-hereditary disease characterized by AVM of the brain and eyes [14]. Osler-Weber-Rendu disease, also known as hereditary hemorrhagic telangiectasia (HHT), is an autosomal-dominant disorder caused by genetic mutations of chromosomes 9 and 12, which result in abnormal angiogenesis and AVM. HHT is diagnosed if three of the Curaçao criteria are present: (1) epistaxis, (2) telangiectasia, (3) AVM in the lungs, liver, brain, spine, or gastrointestinal tract, and (4) family history of HHT in a first-degree relative [12]. AVM may be detected incidentally intra-operatively or in a pathomorphological examination. It could also be encountered unexpectedly on imaging [6]. The symptoms of AVM may relate to the site as in stroke in brain lesions [11], gastrointestinal bleeding or anemia in bowel AVM, mechanical neck syndrome in thyroid AVM [15], vaginal bleeding if located in the uterus [16], abdominal pain, abdominal mass, pulmonary hypertension, high-output heart failure, and embolic phenomena in hepatic lesions [12]. In large lesions, the auscultation may reveal bruits. It may present as a single pathology or be associated with other abnormalities such as arterial aneurysms or malignancy [16]. According to Schobinger’s clinical classification, AVMs have four stages. Stage 1, or the dormant stage, is characterized by erythematous plaques or macules. Stage 2 is marked by the expansion of the lesion. In stage 3, the destruction of the underlying structures occurs. Finally, stage 4 is associated with cardiac decompensation due to high-output cardiac failure [8,17]. Generally, imaging such as plain radiography, CT scan, MRI, or angiography can be used in the diagnosis and assessment of AVMs. MRI is becoming the preferred imaging modality, as it can delineate the lesion size and the extent of invasion, whereas angiography is useful in poorly defined cases and is used as first-stage embolization therapy prior to definitive surgery [18]. Arteriographically, based on origin nidus, AVMs are classified into three groups: 1) arteriovenous fistulae, 2) arteriolovenous fistulae, and 3) arteriolovenulous fistulae [6]. The angio-architecture of AVMs has been categorized into four types according to Cho et al.’s 2006 classification (Figure 3) [17]. 

**Figure 4:**
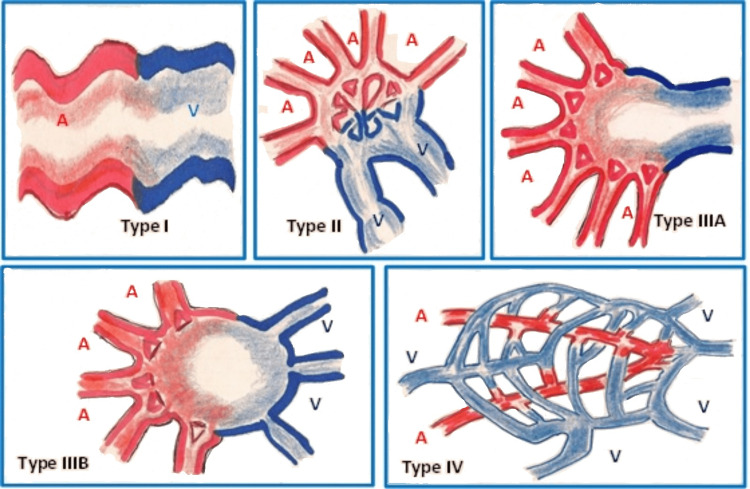
Yakes & Baumgartner's angiographic classification of AVMs AVMs: arteriovenous malformations The illustration has been adapted from Neto et al. (2019) [18]; image redrawn by author Abdalla Saad Abdalla Al-Zawi.

Yakes and Baumgartner, in 2014, suggested another classification for peripheral arteriovenous malformation [18]; this is demonstrated in Figure 4.

Microscopic examination of the lesions will show a large number of vessels of different caliber, including veins and arteries, with veins far more numerous than arteries.

Pathology due to AVM may not affect the physiological function of the endocrine gland. However, there is a chance of functional disorders in the form of either hypo or hyperfunction. Goda et al., in 2021, presented a case of a placental polyp with arteriovenous malformation treated with large doses of human chorionic gonadotropin (hCG) [19]. In our case, we can accept that the symptoms of hyperparathyroidism could be partly related to the AV malformation.

The other issue related to this abnormality is that arteriovenous malformations encountered unexpectedly during surgery may result in remarkable intraoperative bleeding. AVMs are by far the most challenging vascular anomaly to deal with due to the replacement of normal tissue by abnormal vasculature and their very high recurrence rate. Treatment options include minimally invasive surgery with endovascular or percutaneous embolization or open surgical excision. However, a combined approach using both methods is the gold standard approach for treating AVM. Total obliteration of the AVM nidus using an embolization technique results in a better outcome, as this helps reduce the risk of massive bleeding during surgery. In addition to bleeding, the other risk associated with AVM surgery is damage to the host organ. After surgical excision of AVM, local recurrence, especially in the pediatric age group, is not uncommon due to difficulties in achieving radical resection of the lesion or due to the development of a new AVM [7].

## Conclusions

An arteriovenous malformation may be encountered anywhere in the body, and it can alter the function of the hosting organ. We present a unique case of parathyroid adenoma with arteriovenous malformation. This article illustrates the possibility of facing such an unexpected diagnosis and the need to remain vigilant during the management of such a condition.
